# The 40% weight-reduction craze: Market volatility, metabolic nuance, and the quest for sustainable health

**DOI:** 10.1016/j.mocell.2026.100364

**Published:** 2026-05-01

**Authors:** Weiping Han, Peter Shepherd, Kohjiro Ueki, Jia Li, Aimin Xu, Geunho Oh, Jae Bum Kim

**Affiliations:** 1 Institute of Molecular and Cell Biology, A^⁎^STAR Research Enterprise, Singapore; 2 Maurice Wilkins Center for Molecular Biodiscovery, University of Auckland, Auckland, New Zealand; 3 Diabetes Research Center, National Institute of Global Health and Medicine, Japan Institute for Health Security, Tokyo, Japan; 4 Shanghai Institute of Materia Medica, Chinese Academy of Sciences, Shanghai, China; 5 State Key Laboratory of Pharmaceutical Biotechnology, Hong Kong University, Hong Kong; 6 School of Biological Sciences, Seoul National University, Seoul, Korea

**Keywords:** GLP-1, Obesity, Weight loss, Metabolic health, Energy metabolism

## Abstract

The pharmaceutical industry is currently engaged in an intense competition to maximize weight reduction percentages. The evolution from standard glucagon-like peptide-1 (GLP-1) receptor agonists to dual and triple agonists and even more complex combinations has fundamentally shifted clinical benchmarks and investor expectations. We are now witnessing targets of 30%-40% of body mass, a range that could be achieved only through bariatric surgery previously. However, this numerical obsession is starting to decouple the metric of weight loss from the real objective of metabolic health. By focusing purely on total mass reduction, the industry risks sacrificing metabolic integrity and vital physiological components like muscle mass in the pursuit of higher percentages.

## Graphical Abstract

**Figure:**
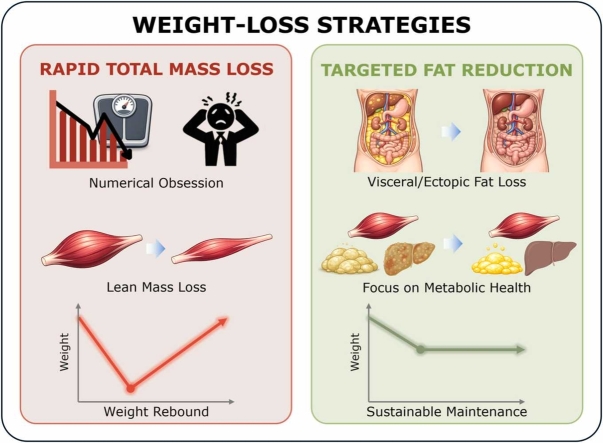

## MARKET VOLATILITY AND THE FINANCIALIZATION OF CLINICAL BENCHMARKS

Market valuations for industry leaders like Eli Lilly and Novo Nordisk have become hyper-sensitive to trial results measured in percentage points. In this high-stakes environment, clinical success is no longer defined by absolute patient improvement but by achieving relative dominance in a concentrated duopoly.

The volatility of this sector was first clearly demonstrated in December 2024. Novo Nordisk shares experienced a massive shock, plunging by as much as 26% initially before stabilizing near a 20% drop. This reaction followed headline results for CagriSema (semaglutide/cagrilintide) in the REDEFINE 1 trial; although the drug achieved an impressive 22.7% weight loss (Garvey et al., 2025), it failed to meet the 25% target that Novo Nordisk had signaled to investors. This single-day sell-off erased approximately 125 billion dollars in market capitalization.

History repeated itself on February 23, 2026, following the release of the REDEFINE 4 Phase 3 results. In this head-to-head study, CagriSema delivered an impressive 23.0% weight loss under ideal conditions but was statistically outperformed by Eli Lilly’s tirzepatide, which achieved 25.5% (Table 1).

**Table 1:** Comparative analysis of efficacy and market valuation impact for Novo Nordisk’s CagriSema and Eli Lilly’s Tirzepatide (February 23, 2026)

**Metric**	**Novo Nordisk**	**Eli Lilly**	**Differential**
	**CagriSema (2.4/2.4 mg)**	**Tirzepatide (15 mg)**	
Adherent weight loss	23.0%	25.5%	2.5%
Real-world (ITT analysis)	20.2%	23.6%	3.4%
24-hour market cap impact	Loss of USD100 Billion	Significant increase	N/A
Baseline mean weight	114.2 kg	114.2 kg	N/A

The table summarizes weight loss outcomes and financial market responses following clinical evaluations of Novo Nordisk’s CagriSema (2.4/2.4 mg) and Eli Lilly’s Tirzepatide (15 mg). Efficacy is presented as percentage weight loss under adherent conditions and Real-World Intent-to-Treat analysis. Market impact is quantified by the 24-hour change in market capitalization following data disclosure. Baseline mean weight for both study cohorts was 114.2 kg.

The resulting 15.1% drop in Novo Nordisk’s stock illustrates a dangerous trend: the market now views a 20% weight reduction as a baseline liability rather than a medical success. This environment pressures firms to chase hyper-potent targets of 30% or 40% loss, even when such goals are unnecessary for the average patient's health.

## THE DANGER OF UNIFORM TARGETS

**Fig. 1:**
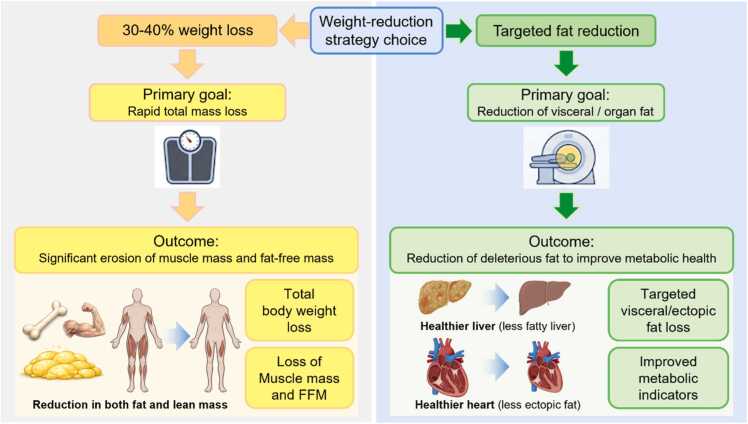
*Divergent outcomes of weight-reduction strategies: total weight loss vs. targeted fat reduction.* The schematic illustrates 2 distinct pathways originating from a "Weight-reduction strategy choice." The left pathway (yellow) highlights the "30%-40% weight loss," which leads to significant erosion of muscle mass and fat-free mass (FFM). The right pathway (green) represents a "targeted fat reduction" approach, prioritizing the mobilization of deleterious visceral and organ-specific ectopic fat to improve metabolic outcomes.

While a 5%-10% weight loss is sufficient to improve standard markers like blood pressure and glycemia for most individuals, specific diseases do require higher weight loss (Fig. 1). Remission of type 2 diabetes and significant improvement in metabolic dysfunction-associated steatohepatitis (MASH) often require losing 15% or more (Lingvay et al., 2022). These high thresholds represent specialized clinical needs and not as universal requirements for the general population seeking metabolic improvement.

## THE BMI PARADOX AND EAST ASIAN METABOLIC HEALTH

The "40% obsession" relies heavily on Body Mass Index (BMI), a metric that fails to distinguish between lean mass and adipose tissue or account for ethnic variations in fat distribution.

East Asian populations frequently manifest a metabolically obese normal-weight phenotype (Ruderman et al., 1998). These individuals often develop metabolic syndrome and type 2 diabetes at BMI levels that Western standards classify as "normal" (18.5-22.9 kg/m^2^) (Table 2).

**Table 2:** Comparison of BMI classifications by population group

**Population group**	**Normal weight (kg/m**^**2**^**)**	**Overweight (kg/m**^**2**^**)**	**Obesity (Class I) (kg/m**^**2**^**)**
Western/Caucasian	18.5-24.9	25.0-29.9	≥30.0
East Asian	18.5-22.9	23.0-24.9	≥25.0

Thresholds for Normal Weight, Overweight, and Obesity (Class I) across Western/Caucasian and East Asian demographics. The distinct cut-offs illustrate the clinical adjustments made to account for ethnic variations in metabolic risk profiles.

At identical BMIs, East Asians typically possess 3%-5% more body fat than Caucasians (Deurenberg et al., 2002). For a patient with a moderate BMI of 24 kg/m^2^, the pursuit of a 30% weight reduction could be physically devastating, leading to severe sarcopenia and other health issues. Conversely, their underlying metabolic issues, such as visceral inflammation, could be addressed with a targeted 3%-5% fat reduction focused specifically on the organ compartments.

## BIOLOGICAL CROSS-TALK: BRAIN-BODY AND INTER-ORGAN MISCOMMUNICATION

To protect metabolic integrity, we must look beyond total mass to the complex biological signals exchanged between the brain and the body, such as fat and muscle.

The hypothalamic "satiety mask": The central nervous system normally uses signals like leptin to monitor energy stores. When weight drops sharply, circulating leptin levels crash, which the brain interprets as a starvation emergency. GLP-1 medications pharmacologically "mask" this signal by dampening the hypothalamic networks that would normally drive hunger (Friedrichsen et al., 2021). This creates a homeostatic mismatch: the person feels satiated even though their internal sensors are screaming for nutrients.

Adipocyte-muscle dysfunction and "iatrogenic undernutrition": Visceral fat acts as a rogue endocrine organ, releasing pro-inflammatory cytokines that can increase myostatin—a potent inhibitor of muscle growth—by up to 100-fold (Allen et al., 2008). Furthermore, potent GLP-1 therapy suppresses glucagon so effectively that it can impair the liver's ability to clear amino acids (Tippairote et al., 2026). When combined with the reduced protein intake caused by appetite suppression, the body enters a state of undernutrition. Even as the brain signals the body to stop eating, the lack of metabolic coordination forces skeletal muscle to supply essential amino acids through proteolysis.

## MUSCLE INTEGRITY: QUANTITATIVE LOSS VS FUNCTIONAL CHANGE

In trials such as SUSTAIN 8, over 40% of the weight lost was fat-free mass, which includes muscle, bone, and water (McCrimmon et al., 2020). While some high-BMI patients may see improved physical function because their absolute fat burden is reduced, this is not a universal benefit. For East Asians with moderate BMIs, the absolute loss of lean mass is a primary risk for long-term functional decline.

Recognizing the importance of muscle mass in long-term health, the pharmaceutical industry has been actively developing muscle-preserving and -improving treatments. For example, in a phase II trial conducted by Eli Lilly, adding bimagrumab, an experimental human monoclonal antibody targeting type II activin receptors, to semaglutide resulted in a 22.1% weight loss, with nearly 93% of that reduction coming from fat, effectively preserving muscle mass (Heymsfield et al., 2026). Another example is the COURAGE2 trial conducted by Regeneron. Compounds like trevogrumab (anti-myostatin) and garetosmab (anti-activin A) have been shown to reduce lean mass loss by up to 80% (https://investor.regeneron.com/news-releases/news-release-details/results-phase-2-courage-trial-demonstrating-potential-improve/).

## THE REBOUND EFFECT

Weight regain is a major challenge, occurring at a rate of approximately 0.8 kg per month after stopping treatments like semaglutide and tirzepatide (West et al., 2026). The same *BMJ* meta-analysis confirmed that stopping these medications leads to a rapid loss of health benefits. Blood pressure, cholesterol, and glycated hemoglobin (HbA1c) levels return to their original pathological baselines often within just 12-14 months (West et al., 2026).

## SAFETY AND BIASED AGONISM: A DUAL RISK

As GLP-1 therapy is extended to younger patients, concerns about the long-term impact of chronic receptor activation on the developing brain have intensified.

**Fig. 2:**
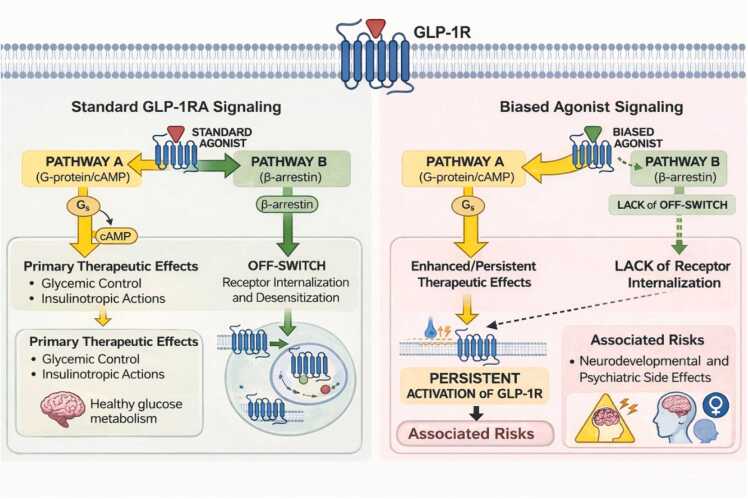
*Signal biasing of GLP-1 receptor agonism and associated downstream effects.* The schematic compares the signaling profiles of standard GLP-1 receptor agonists (GLP-1RA) versus biased agonists. Pathway A (G-protein/cAMP) mediates the primary therapeutic effects, including glycemic control and insulinotropic actions. Pathway B (β-arrestin) traditionally serves as an "off-switch" through receptor internalization and desensitization. Biased agonists are shown to selectively favor Pathway A, with loss of off-switch in Pathway B, resulting in persistent activation of GLP-1R, which may be associated with neurodevelopmental and psychiatric side effects.

A new direction in drug development involves "biased" or "β-arrestin sparing" agonists (e.g., orforglipron, CT-868, PF-08653944) (Chakravarthy et al., 2026; Rosenstock et al., 2025; https://www.pfizer.com/news/press-release/press-release-detail/pfizers-ultra-long-acting-injectable-glp-1-ra-shows-robust). Standard agonists trigger both G-protein pathways (for metabolic control) and β-arrestin pathways (the receptor's "off-switch" that leads to internalization). Biased agonists skip the β-arrestin "off-switch." While this reduces gastrointestinal side effects like nausea, it keeps receptors active on the cell surface for longer periods. By bypassing the natural internalization process, these drugs may subject the brain to continuous activation of neural pathways, potentially amplifying neurodevelopmental and psychiatric risks (Fig. 2).

## CONCLUSION: TOWARD A SUSTAINABLE PARADIGM

The current 40% weight loss craze is largely driven by market pressures rather than clinical necessity. By shifting our focus from total mass to visceral and organ fat reduction, we can move away from the cycle of rapid regain and toward a sustainable model of lifelong metabolic health. This transition requires that we stop treating weight as a singular metric and start focusing on metabolic health by treating weight as a complex biological variable influenced by ethnicity and body composition.

## Funding and Support

This work was supported by the National Research Foundation of Korea (NRF) grant funded by the Korean government (RS-2020-NR049402, RS-2023–00218616, and RS-2025–02214748 to J.B.K.). Research in the Laboratory of W.H. is also supported by A*STAR intramural and national research programs.

## Author Contributions

All authors contributed to wrote the manuscript. G.O., W.H., and J.B.K. edited the manuscript.

## Declaration of Competing Interest

The authors declare that they have no known competing financial interests or personal relationships that could have appeared to influence the work reported in this paper.
